# Association between Urinary AGEs and Circulating miRNAs in Children and Adolescents with Overweight and Obesity from the Italian I.Family Cohort: A Pilot Study

**DOI:** 10.3390/jcm12165362

**Published:** 2023-08-18

**Authors:** Paola Russo, Fabio Lauria, Ivana Sirangelo, Alfonso Siani, Giuseppe Iacomino

**Affiliations:** 1 Institute of Food Sciences, National Research Council, Via Roma 64, 83100 Avellino, Italy; prusso@isa.cnr.it (P.R.); alfonso.siani@isa.cnr.it (A.S.); giuseppe.iacomino@isa.cnr.it (G.I.); 2 Department of Precision Medicine, School of Medicine and Surgery, University of Campania “Luigi Vanvitelli”, S. Andrea Delle Dame-Via L. De Crecchio 7, 80138 Naples, Italy; ivana.sirangelo@unicampania.it

**Keywords:** circulating miRNAs, urinary AGEs, disease propensity biomarkers, obesity, children and adolescents

## Abstract

Modern dietary habits are linked to high exposure to Advanced Glycation End products (AGEs) mainly due to the dramatic increase in the consumption of highly processed foods in recent years. Body levels of these compounds vary with food intake and are almost interconnected with age and health status, formally embodying indicators of oxidative stress and inflammation in adults. However, the relationship between AGEs and health issues has not been definitively understood in children, and several pediatric investigations have produced conflicting evidence. Besides, despite extensive research, there are no universally accepted analytical techniques for measuring AGE levels in the human body, with several approaches available, each with its advantages and disadvantages. This pilot study aimed to investigate the association between urinary AGEs, measured using spectrofluorimetry-based assays, and circulating microRNAs (c-miRNAs) in a subsample (*n* = 22) of Italian children participating in the I.Family Study. Anthropometric measurements, biochemical markers, and miRNA profiles were assessed. The first indication of a relationship between urinary AGEs and c-miRNAs in the context of obesity was found. Specifically, four miRNAs, hsa-miR-10b-5p, hsa-miR-501-5p, hsa-miR-874-3p, and hsa-miR-2355-5p were significantly associated with levels of urinary AGEs. The association between AGEs, obesity, inflammation markers, and specific miRNAs highlights the complex interplay between these factors and their potential impact on cellular and tissue homeostasis. The discovery of altered c-miRNAs profiling has the potential to offer innovative methods for assessing early changes in the body’s AGE pool and allow recognition of an increased risk of disease susceptibility, routinely undetected until metabolic complications are identified.

## 1. Introduction

The reaction between the free amine groups of proteins, lipids or nucleic acids and the carbonyl group of reducing sugars generates a variety of biochemical substances known as AGEs (Advanced Glycation End products). AGEs can be derived exogenously from diet or generated endogenously in the body. The formation of dietary AGEs (dAGEs) is the consequence of a series of multistep nonenzymatic reactions first described by Louis Camille Maillard in 1912 [1]. Due to the high variety of food ingredients and the processing at high temperatures, the Western diet is considered constitutively rich in dAGEs [2,3]. Remarkably, these compounds are only partially cleared after nutritional intake and are to some extent absorbed in the gastrointestinal tract before entering into the bloodstream, accumulating in the tissues, and increasing the systemic AGE load [4,5]. Of note, the byproducts of the Maillard reaction have been characterized as being continuously formed also under normal conditions in vivo [6], and are generated more quickly in response to stress, as well as oxidative stress and hyperglycemia, directly depending on blood glucose levels, or as a byproduct of lipid peroxidation [7]. AGE levels are commonly low in healthy and young subjects but gradually increase with aging: their accumulation in tissues and on vulnerable plasma proteins throughout life is considered a main contributing factor to the typical physiological decline associated with aging, as it is directly involved in the pathogenesis of musculoskeletal degenerative diseases in the elderly [8,9]. Since the occurrence and storage of AGEs in many cell types affect intracellular and extracellular organization and functions, epidemiological studies have demonstrated a connection between higher body levels of AGEs and an increased risk for cardiovascular disease [10,11], liver and kidney disease [12], neurodegenerative diseases [13], complications from diabetes [7,14], bone and muscle diseases [15], mental health conditions [16], and others. The basic process by which these compounds are interconnected to the development of this variety of disorders is not completely understood. However, AGEs have been suggested to have a role as predictors of low-intensity chronic inflammation, in the upturn of the innate immune system, and oxidative stress [17]. Every tissue in the body can be affected by AGEs, either by direct cellular damage through protein cross-linking or by interaction with cell surface receptors [18]. Of note, the interaction between AGEs with RAGE, their most well-known cell surface receptor, promotes a cascade of intracellular signals that activates the nuclear transcription factor kappa-B (NF-κB) pathway and NADPH oxidase, thereby supporting oxidative stress and inducing inflammation [19]. Exogenous intake, endogenous generation, renal and fecal excretion, and enzymatic clearance all affect AGE levels in the bloodstream. Depending on the correlation between the intake of dAGEs and their levels in body fluids, these compounds could represent useful nutritional biomarkers.

Despite extensive investigation linking AGEs to a wide range of clinical conditions, there are no universally accepted analytical techniques for measuring their levels in the human body; several approaches are available, each with its benefits and drawbacks [20]. As well, these compounds can be divided into different groups according to their chemical makeup and fluorescence-emitting capabilities. According to a recent review [20], AGE-specific fluorescence is increasingly used for detecting the buildup of fluorescent AGEs in human fluids and tissues since the majority of discovered AGEs are characterized by excitation/emission wavelengths at 370 nm and 440 nm, respectively. In this context, the use of spectrofluorimetry-based assays is both simple and cost-effective as a test to determine AGE levels in population screening.

MicroRNAs (miRNAs) are small noncoding RNAs with a length of 20–24 nucleotides that control gene expression post-transcriptionally [21]. A unique miRNA species can regulate one or more transcripts at once (up to hundreds) while a single mRNA frequently displays several interaction sites for different miRNAs, leading to the setting up of complex regulatory circuits [22]. These molecules are expressed in a variety of organs and cells and can influence practically all cell processes. The mounting data highlight their value as consistent, non-invasive, and trustworthy biomarkers for a variety of pathophysiological diseases [23,24]. Most research has focused on miRNAs as biomarkers in cancer research [25], emphasizing their relevance as personalized theranostic factors [26]. Besides, many papers have identified correlations between altered levels of circulating miRNAs (c-miRNAs) and the physiopathology of numerous processes, including mitochondrial [27], cardiovascular [28], neurodegenerative [29], immune disease [30], metabolic [23,24,31], rare genetic disorders, and more. Specifically, changes in miRNA profiles have been linked to obesity and other non-communicable disorders in numerous studies. However, the bulk of the functional significance of c-miRNAs still seems to be unknown as of right now, and the gap between discovery and function has not been closed. miRNAs have also been recognized as key biochemical players in affecting the inflammatory response since they are epigenetic players that regulate immune cell growth, immunological response, autoimmunity, and inflammation [32]. Emerging literature shows that the so-called immune-miRs influence both the innate and adaptive immune responses in both health and disease [33,34]. This is because they modulate specific signaling networks in the immune system, and during the inflammatory process the different cells involved undergo significant transcriptional activation that highlights different targets controlled by diverse miRNAs.

In previous studies, we recognized specific c-miRNAs associated with obesity in a subsample of the I.Family study [35], among which hsa-miR-10b-5p was found to be also associated with inflammation [36]. Furthermore, we showed that urinary levels of AGEs are associated with low-grade inflammation in children and adolescents [37]. Thus, the present pilot analysis aims to assess the association of candidate miRNAs with urinary levels of AGEs in apparently healthy children/adolescents from the Italian cohort of the I.Family project, looking for prospective biomarkers for early AGE-induced damage.

## 2. Materials and Methods

### 2.1. Participants

The study was conducted on an Italian subgroup of children/adolescents belonging to the I.Family study, an EC-funded project aiming at investigating determinants of food choice, lifestyle, and related health outcomes in European children and adolescents. A comprehensive description of the I.Family study (registration number ISRCTN62310987) has been published earlier [38]. The study selection criteria and participants’ characteristics, from which the subsample data are drawn out, have been previously reported [39,40]. We included subjects who retained overweight or obesity, i.e., who had a BMI z-score of more than +1 at baseline and after two years at the follow-up, respectively, and did not change more than ±0.1 in BMI z-score per year (defined as overweight/obese, OW/Ob), and subjects who retained normal weight, i.e., who showed a BMI z-score between -1 and +1 at baseline and follow-up and did not change more than ± 0.1 in BMI z-score per year (defined as normal weight, NW) [41]. Current investigation aimed to assess the association of candidate miRNAs with levels of urinary AGEs in 22 children/adolescents of which anthropometric information and levels of high-sensitivity C-reactive protein (hs-CRP) were available. Approval by the national ethics committees was obtained by the local Health Authority (ASL Avellino). The study was conducted according to the principles set by the Declaration of Helsinki.

### 2.2. Anthropometric Measurements

A detailed description of the anthropometric measurements in the I.Family project, including intra- and inter-observer reliability, has been published elsewhere [42]. Briefly, weight was determined to the nearest 0.1 kg using a body composition analyzer (Tanita BC420 SMA, Tanita Europe GmbH, Sindelfingen, Germany) with participants in fasting status, without shoes and with light clothing. Height was measured with a calibrated stadiometer (Seca 225, Seca GmbH & Co., KG., Hamburg, Germany) and recorded to the nearest 0.1 cm. BMI was calculated by dividing body weight (in kg) by height squared (in m^2^). Age- and sex-specific BMI z-scores were calculated according to Cole and Lobstein [41].

### 2.3. Biochemical Analysis and c-miRNAs Profiling

According to standard protocols, the fasting venous blood samples were collected in BD Vacutainer^®^ blood tubes. An earlier publication provided a thorough explanation of the sample collection and biochemical analysis methods [43]. Chemical-clinical analyses were done as part of routine laboratory testing, in a central laboratory (the University of Bremen, Centre for Biomolecular Interactions Bremen-CBIB). Serum samples stored at −80 °C were used to detect levels of hs-CRP, (using either single or MULTI-SPOT^®^ Assay Systems, Meso Scale Discovery, Rockville, MD, USA).

Taking advantage of the qPCR array technology, we previously determined c-miRNA profiles in children and adolescents of the I.Family study [40]. Methods for miRNA extraction and screening from plasma samples have been previously published [35,40]. Individual plasma samples were first tested for hemoglobin levels and hemolyzed samples were omitted from the analysis [35]. Different assays were performed in triplicate by using the miScript Primer Assays according to the manufacturer’s instructions (Qiagen, Hilden, Germany). Relative miRNA levels were normalized using the endogenous spike-in Cel-miR-39 [35] employing the Data Assist v3.1 software package (Life Technologies, Thermo Fisher Scientific, Milan, Italy).

### 2.4. Urinary Fluorescent-AGEs Detection

Children and adolescents collected the morning urine samples at home and brought them to the study center the same day. Parents were instructed to preserve the sample in the refrigerator at home if the time interval between collection and delivery exceeded two hours. At the study center, urine samples were stored at −80 °C on the same day of collection. A Perkin Elmer Life-Sciences LS 55 spectrofluorimeter was used to measure the urinary fluorescent AGEs. Fluorescence spectra were captured between 400 nm and 600 nm, upon excitation at 370 nm, at room temperature, using urine samples diluted 1:10 in phosphate-buffered saline [37]. The fluorescence intensity was adjusted to correspond to the emission maximum, which is centered at 440 nm, by removing the background. The relative fluorescence intensity (reported in arbitrary units, AU) was adjusted for the urinary creatinine concentration calculated as g/L since the urinary AGE concentration relies on the urine volume. Creatinine in urine was determined by Jaffe’s reaction (COBAS INTEGRA 400 plus, Roche Diagnostics Ltd., Rotkreuz, Switzerland).

### 2.5. Statistical Analysis

Statistical analyses were achieved by using IBM SPSS Statistics software (v28.0.1.0, IBM Corp., Armonk, NY, USA). Analyses were performed separately for NW and OW/Ob subjects. Data collected were calculated as means and 95% confidence intervals (CIs). Associations of miRNA expression with urinary AGEs were assessed using linear regression analyses through two different models. Generated models were adjusted for covariates including age, sex, BMI z-score (model I), and covariates of the model I + hs-CRP (model II). The Benjamini–Hochberg (BH) procedure was adopted to control the false discovery rate (FDR). The level of statistical significance was set at α < 0.05.

## 3. Results

The anthropometric and biochemical markers of the 22 participants, divided into NW (*n* = 10) and OW/Ob (*n* = 12), are reported in Table 1. Of note, no one of the study subjects suffered from metabolic syndrome or diabetes. Regarding levels of urinary AGEs, a minor difference was observed between NW and OW/Ob children.

In the current study, after plasma extraction, the candidate miRNAs were determined in individual assays by RT-qPCR. Differences in miRNA signatures with respect to anthropometric and biochemical variables were analyzed separately in NW and OW/Ob children/adolescents. Results reported in Table 2 are adjusted for covariates. AGE levels show a significant association with hsa-miR-10b-5p (MIMAT0000254), hsa-miR-501-5p (MIMAT0002872), hsa-miR-874-3p (MIMAT0004911), hsa-miR-2355-5p (MIMAT0016895) only in OW/Ob subjects. With the exclusion of hsa-miR-10b-5p, no association of candidate miRNAs with hs-CRP was found.

## 4. Discussion

Modern dietary habits are connected with high exposure to AGEs since the consumption of high-processed foods as well as sugars and fats has dramatically risen over the past years. According to a large body of evidence, levels of these compounds in plasma, urine, and saliva vary with food intake and are nearly interconnected with age and health issues, formally embodying indicators of oxidative stress and inflammation. The relationship between AGEs, inflammation, and health status is not definitively understood in children, and various pediatric investigations have produced contradictory data. In a recent paper, we demonstrated that AGEs-linked urinary fluorescence is positively associated with markers of sub-clinical inflammation as well as C-reactive protein level in an Italian population of otherwise healthy children and adolescents [37], confirming that AGEs may have a role in the process of subclinical inflammation.

In previous studies, we showed characteristic c-miRNA profiles associated with health status in a cohort of children and adolescents of the I.Family study [40]. In the current research, we investigated the conceivable association of AGEs with candidate miRNAs in a subsample of healthy Italian children and adolescents of the I.Family study cohort for whom AGE levels had also been determined. NW and OW/Ob subjects were separated in the statistical analysis since a very close association between dietary AGEs and the amount of food or calories ingested has been earlier reported [44]. Interestingly, we found the first indication pointing toward a relationship between levels of AGEs and c-miRNAs in the context of obesity. Of note, miRNAs can be affected by the accumulation of AGEs and/or can modulate the expression of genes involved in AGEs formation or degradation pathways. Among the screened, four miRNAs, hsa-miR-10b-5p, hsa-miR-501-5p, hsa-miR-874-3p, and hsa-miR-2355-5p, were associated with AGEs levels in the statistical analysis only in OW/Ob children/adolescents. In this group, the inclusion of hs-CRP as a covariate did not lower the statistical significance. Remarkably, we previously recognized that hsa-miR-10b-5p was associated with overweight/obesity [35]. We also found an association between circulating hsa-miR-10b-5p and plasma hs-CRP protein in children with overweight and obesity [36]. Together, these data point towards a cross-talk among levels of AGEs, obesity, biomarkers of inflammation, and specific miRNAs even in children. Overall, preliminary results underscore the close link between AGE levels and miRNA dysregulation, an interplay that may affect cellular and tissue homeostasis and related health problems. However, the relationship between miRNAs, AGEs, and inflammation is complex and requires further research to fully understand the underlying mechanisms. Besides, it is not defined whether the miRNAs found associated with urinary AGEs are the drivers of metabolic changes related to AGE exposure or represent epiphenomena. A deeper understanding of the key factors influencing this communication could contribute to a better comprehension of the mechanisms of AGEs induced subclinical inflammation, even in pediatric age. The results look exciting considering the complexity of testing in vivo the heterogeneous class of AGEs, the levels of which are generally low in young healthy subjects but gradually increase with aging contributing to harmful outcomes related to health status, and life expectancy.

This pilot study has some strengths and limitations. First, the main strength is the fact that this is the first research linking c-miRNAs with urinary AGE levels in healthy young people for potential biomedical applications, whereas to date only a limited number of studies have been directed at identifying miRNAs altered by AGE stimulation in cellular and animal models [45,46]. Of note, the detection of c-miRNAs could be useful both for the study of the etiopathogenetic mechanisms induced by AGEs and as perspective biomarkers of disease propensity, disease state, and/or responsiveness to dietary interventions even in the pediatric age group. This aspect is relevant also considering that therapeutic strategies to decrease the levels and effects of AGEs remain limited to date. The main drawback of the present study is the small sample size. In addition, the use of spectrofluorimetry-based assays for AGE detection, although fast and inexpensive, allows the determination of fluorescent AGEs in urine without specifying the type of AGE evaluated; besides, the method does not allow the detection of non-fluorescent AGE. Therefore, more research and large-scale genetic studies are needed to assess the role of the identified miRNAs. In addition, the mechanism by which AGEs affect miRNA expression was not determined. These will be the subject of future studies.

## 5. Conclusions

The contribution of AGEs to the inflammatory state and the pathogenesis of many diseases has traditionally been documented in chronic age-related inflammatory disorders [18]. In this feasibility study, we sought to shed light on the potential relationship between c-miRNAs and increased urinary AGEs in children and adolescents with overweight and obesity. Interestingly, the discovery of an altered profile of c-miRNAs would have the potential to offer innovative methods to assess variations in the body AGE pool in early stages and recognize an increased risk of disease propensity, habitually undetected until metabolic complications are identified. However, confirmatory studies are required given the small sample sizes and the lack of long-term outcome data. Further studies are expected to identify and validate miRNAs differentially expressed in early AGE-induced dysfunction, thus requiring more attention in this largely unexplored area.

## Figures and Tables

**Table 1:** Anthropometric and biochemical characteristics of the study sample.

	NW (*n* = 10, Male = 8)	OW/Ob (*n* = 12, Male = 4)
Age	12.05 ± 1.25	12.23 ± 1.38
BMI z-score	0.18 ± 0.40	1.97 ± 0.77
Urinary AGEs (AU)	241.0 ± 55.4	333 ± 134.8
hs-CRP (mg/dL)	0.11 ± 0.13	0.42 ± 0.67
hsa-miR-10b-5p	2.18 ± 1.39	2.37 ± 1.23
hsa-miR-501-5p	0.47 ± 0.17	0.80 ± 0.49
hsa-miR-874-3p	4.80 ± 1.75	4.60 ± 2.73
hsa-miR-2355-5p	0.50 ± 0.41	0.59 ± 0.34

Data are expressed as mean (CIs) or as frequency (%). BMI: Body Mass Index; AGEs: Advanced Glycation End products; hs-CRP: high-sensitivity C-Reactive Protein; hsa-miR: Homo sapiens (human) micro RNA.

**Table 2:** Association of c-miRNA levels with urinary AGEs in children/adolescents.

	NW (*n* = 10)			OW/Ob (*n* = 12)		
	B	*p*-Values	*q*-Values	B	*p*-Values	*q*-Values
hsa-miR-10b-5p						
I model	−0.57 (−0.28–0.17)	0.541	0.974	0.27 (0.09–0.45)	**0.009**	**0.012**
II model	−0.14 (−0.38–0.10)	0.187	0.622	0.27 (0.06–0.48)	**0.020**	**0.022**
hsa-miR-501-5p						
I model	−0.73 (−2.45–0.99)	0.323	0.974	0.52 (0.15–0.90)	**0.013**	**0.013**
II model	−0.77 (−2.62–1.08)	0.311	0.622	0.51 (0.11–0.92)	**0.022**	**0.022**
hsa-miR-874-3p						
I model	0.01 (0.09–0.77)	0.774	0.974	0.10 (0.04–0.16)	**0.007**	**0.012**
II model	−0.03 (−0.23–0.17)	0.714	0.952	0.10 (0.03–0.18)	**0.015**	**0.022**
hsa-miR-2355-5p						
I model	−0.01 (−0.75–0.73)	0.974	0.974	−1.26 (−2.08–0.43)	**0.009**	**0.012**
II model	−0.02 (−0.84–0.80)	0.952	0.952	−1.27 (−2.79–0.47)	**0.008**	**0.022**

Data are Beta values (CIs). Covariates: (model I) Age, sex, BMI z-score; (model II) model I + hs-CRP. *q*-values are BH-adjusted *p*-values. Values in bold indicate statistically significant results. hsa-miR: Homo sapiens (human) micro RNA.

## Data Availability

All data produced or analyzed during this study are included in this article.
